# Supplementary material for: Deciphering genetic causes for sex differences in human health through drug metabolism and transporter genes
Source: Nat Commun. 2023 Jan 12;14:175. doi: 10.1038/s41467-023-35808-6 (PMC9837057; doi:10.1038/s41467-023-35808-6)
Supplement: Supplementary file 1 — Supplementary Information [file 41467_2023_35808_MOESM1_ESM.pdf]

Supplementary information

Supplemental Text for “**Deciphering genetic underlying causes for sex differences in human health through the lens of drug metabolism and transporter genes**”

Yingbo Huang, Yuting Shan, Weijie Zhang, Adam M. Lee, Feng Li1, Barbara E. Stranger, R. Stephanie Huang

This file contains supplemental figures, results, discussion. In general, the text in this document simply expands on findings and discussions from the main text without introducing entirely new findings or discussion topics.

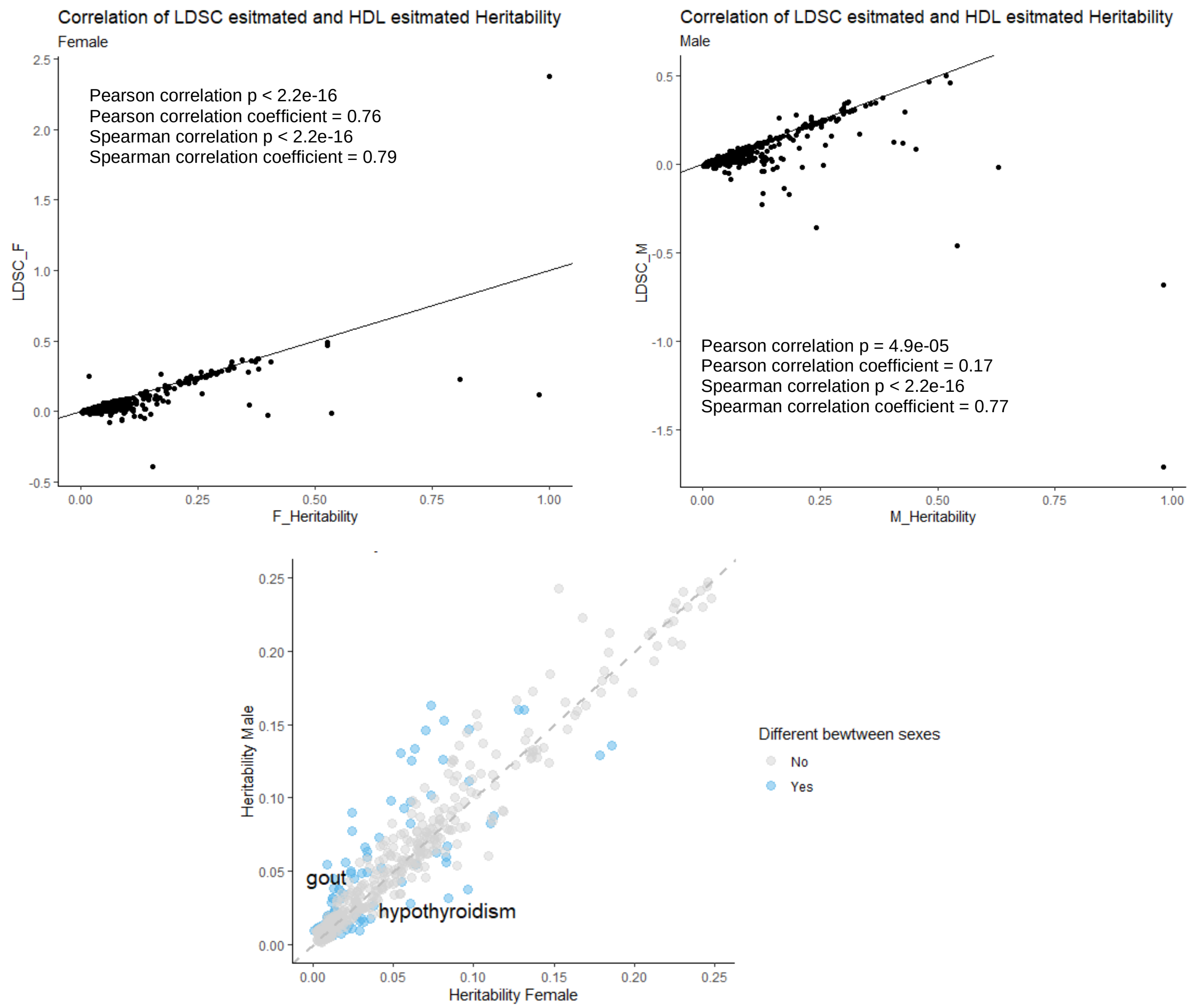

Supplementary Fig. 1. (Top)Correlation of estimated heritability between LDSC method and HDL method in 564 traits. We further verified our results by comparing the estimated heritability using our method with using the linkage disequilibrium (LD) score regression. We observed significant correlation in both males ($P < 2.2 \times 10^{-16}$, cor = 0.77) and females ($P < 2.2 \times 10^{-16}$, cor = 0.79) using the spearman correlation tests. Each dot represents a trait. The LDSC estimated heritability was obtained from Neal's lab (Method). Pearson correlation and Spearman correlation were calculated. (Bottom) Sex differences of estimated heritability in males and in females. Each point represents the estimated heritability for a given trait. This plot is in company

with **Fig. 1B**, with axis ranged from 0 to 0.25. Self-reported Gout and Hypothyroidism were labeled in the figure. (z-score test, two-side, FDR < 0.05).

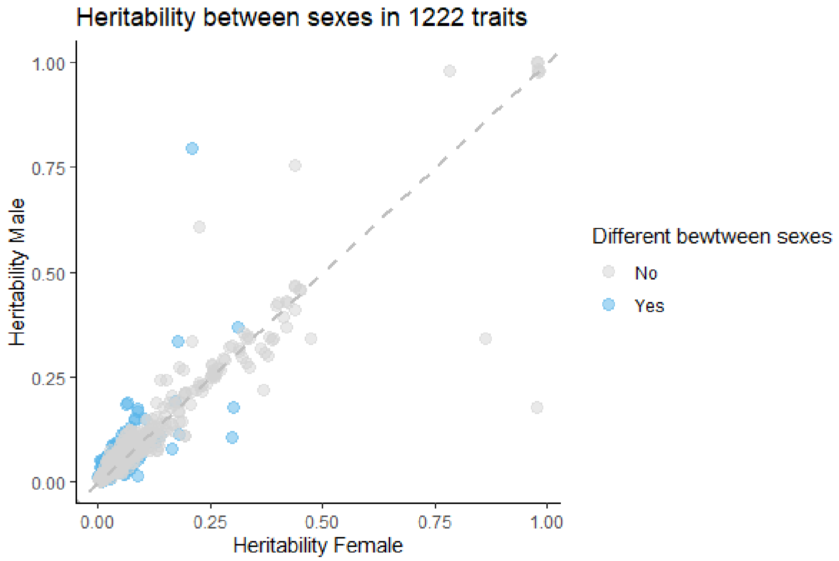

Supplementary Fig. 2. Sex differences of estimated heritability in males and in females for 1222 non-DMET traits in UKBB. Each point represents the estimated heritability for a given trait. We found 13.7% (167/1222) of these traits showing sex differences in global heritability.

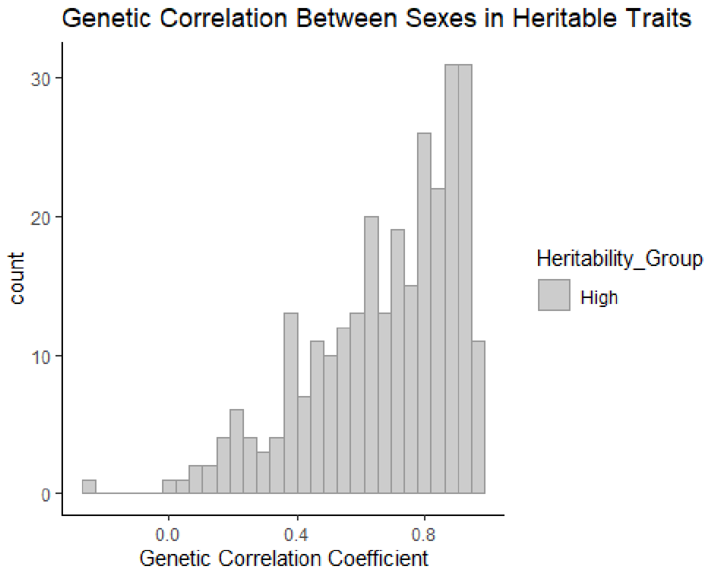

Supplementary Fig. 3. Distribution of genetic correlation coefficient. Histogram plot of genetic correlation co-efficient of the same trait between sexes in 282 heritable traits. After obtaining the genetic correlation of 282 heritable traits using the "*HDL*" package*(60)*, there are 253 traits that have the correlation coefficient differ with 1 (Method) (FDR < 0.05).

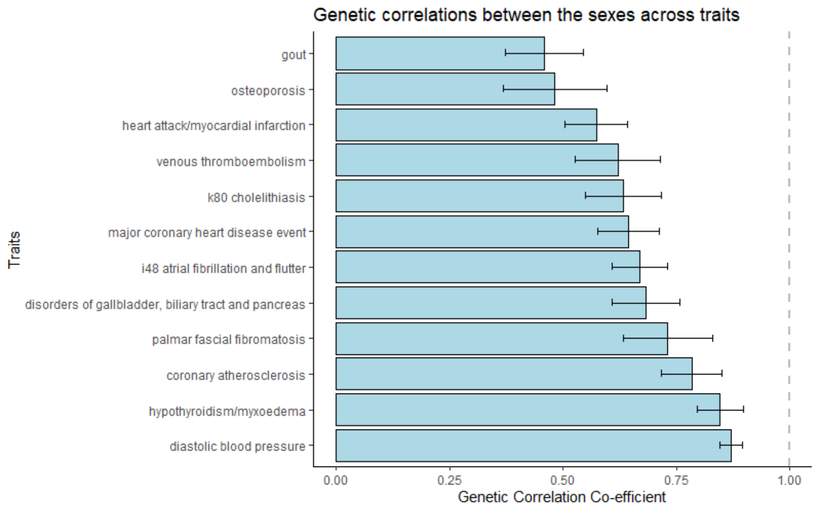

Supplementary Fig. 4. Genetic correlations between sexes across traits. Barplot of the genetic correlation coefficient between the sexes for 12 traits. Black bars indicate the s.e. The centre indicates the value of genetic correlation coefficient.

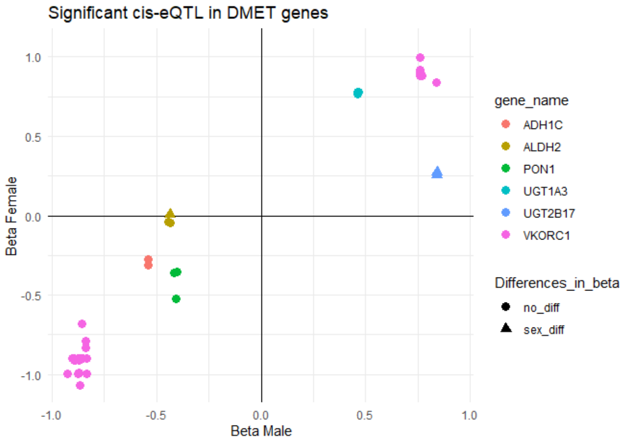

Supplementary Fig. 5. Scatterplot of effect-size of sex-stratified cis-eQTL. Each dot represents a genotype-expression association that meet the statistically significant threshold (FDR < 0.1). The shape indicates whether a sex difference be observed in effect-size. The color denotes the cis gene of the variant. Apart from sex-specific eQTLs, we detected no differences on the effect-size of other significant cis-eQTLs (FDR < 0.1).

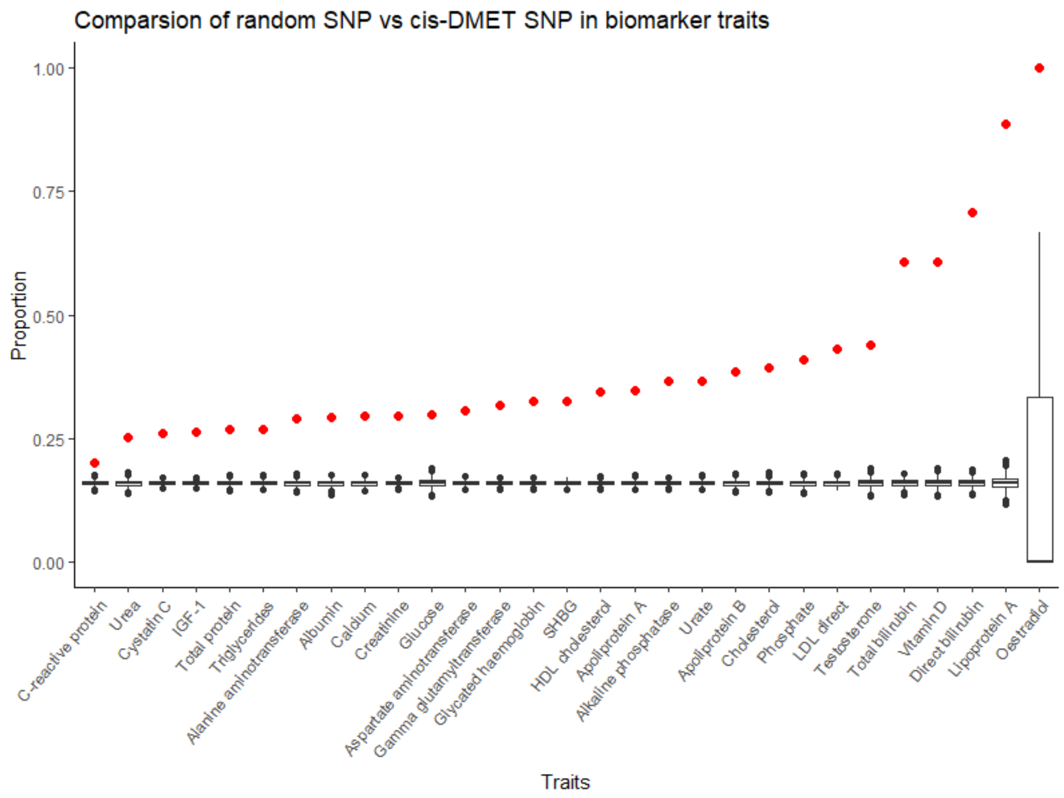

Supplementary Fig. 6. Proportion of SNPs mapped into significant loci in 29 serum biomarkers traits. Boxplot shows the proportion of random selected SNPs (n = 1000 times) that are significant variants ($P < 5 \times 10^{-8}$) in the sex combined serum biomarker traits. The red dots represent the proportion of cis-DMET SNPs that are significant variants. The center line of box plots in show the median, bounds of box represents the first and third quartile, whiskers extend to the minimum and maximum values within 1.5 times of the interquartile range.

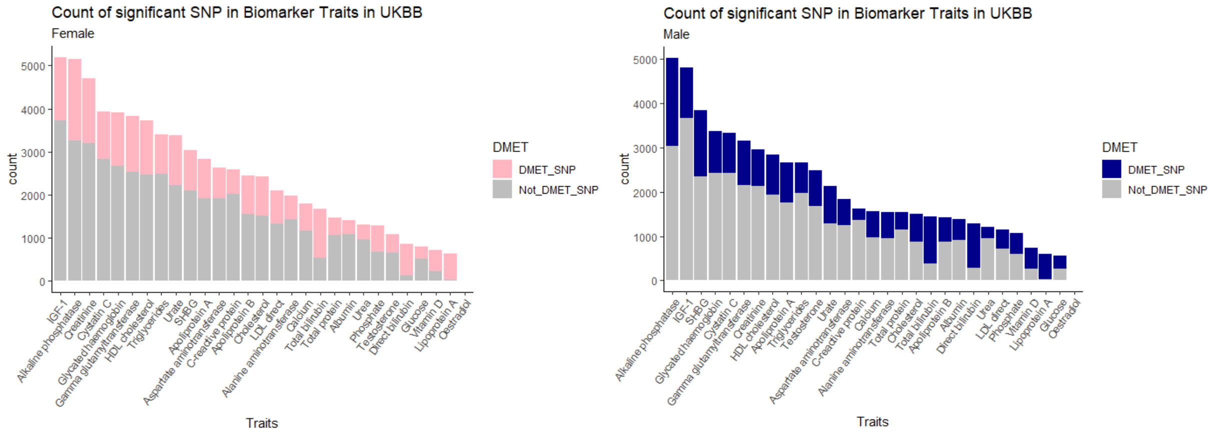

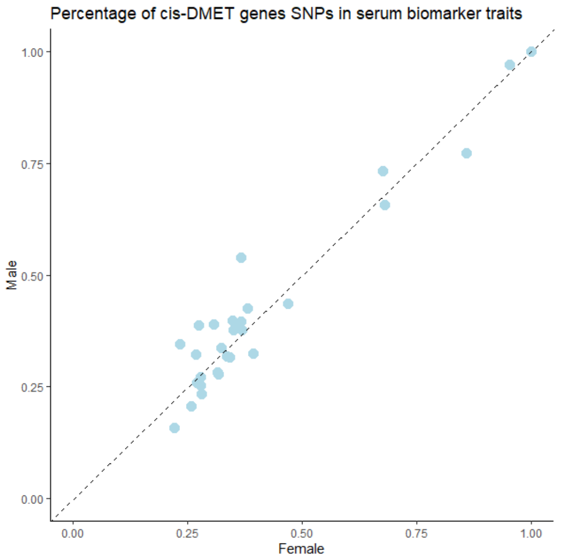

Supplementary Fig. 7. Count of significant variants in the blood biomarker traits. (Top) Barplot shows the total count of significant variants ($P < 5 \times 10^{-8}$) in the sex stratified biomarker traits. Blue/pink color represents variants mapped into the region of the cis-DMET gene. (Bottom) Correlation of the percentage of significant variants are mapped into DMET gene region in serum biomarker traits between sexes.

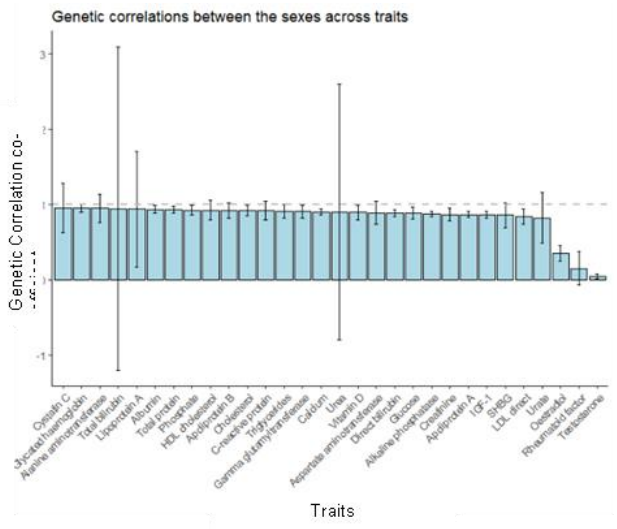

Supplementary Fig. 8. Genetic correlation coefficient of 30 blood biomarker traits in UK Biobank. Barplot of the genetic correlation coefficient between the sexes for 30 traits. Black bars indicate the s.e. We observed 8 traits that showed sex differences in the genetic correlation coefficients. The most significant difference was observed in testosterone (FDR = 6.79×10^{-186}, rg = 0.042), followed by oestradiol (FDR = 3.08×10^{-9}, rg = 0.10), and rheumatoid factor (FDR = 1.11×10^{-3}, rg = 0.15). The centre indicate the value of genetic correlation coefficient.

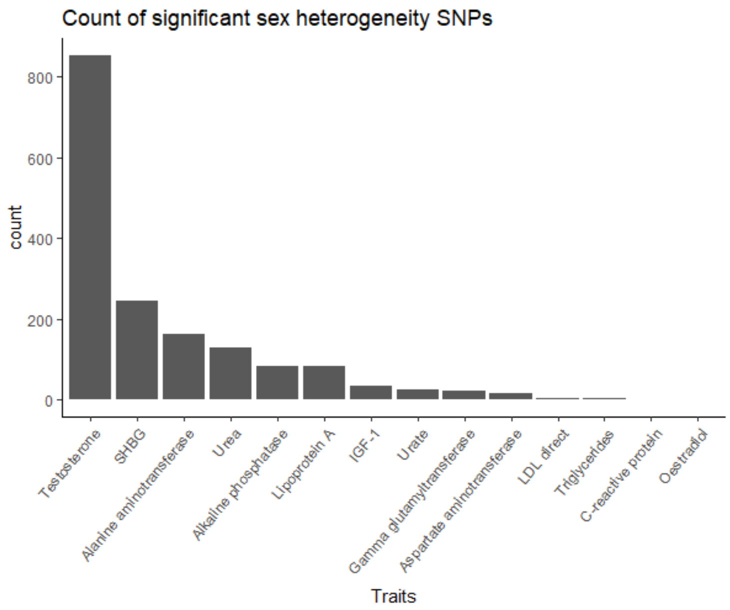

Supplementary Fig. 9. Count of sex heterogeneous variants in cis-DMET genes region in serum biomarker traits. Traits with at least one SNP that presented a statistically significant difference in the genetic effect on traits between sexes are shown. In total, we identified 14 traits that harbor at least 1 sex heterogeneous SNP (**Supplementary Data. 10**) with a total of 1446 sex heterogeneous SNPs. Testosterone harbored the most sex heterogeneous SNPs, which are located in the cis region of DMET genes such as *CYP11B1*, *SLC16A13*, *SLC16A1*, etc.

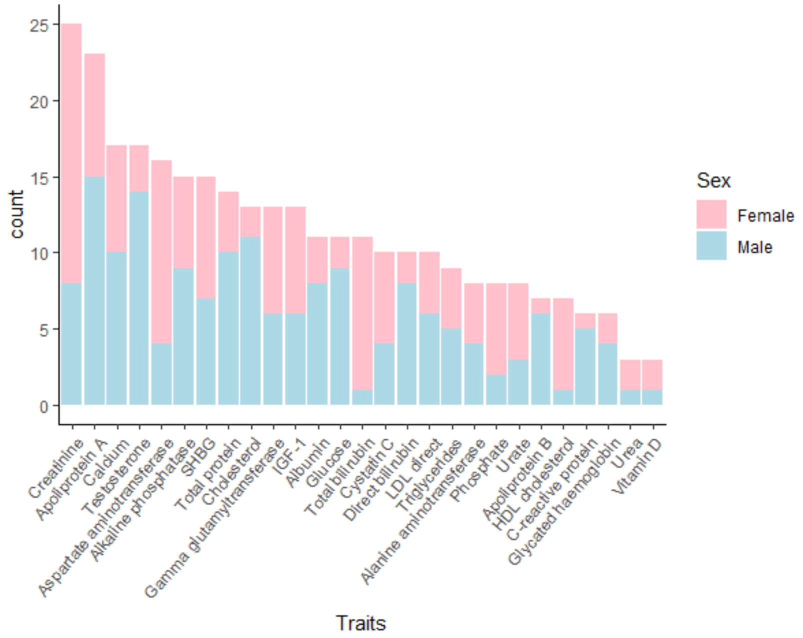

Supplementary Fig. 10. Count of serum biomarker traits that were identified with sex-specific causal relationships in MR analysis. Blue/pink color represents relationships either specific in male or female.

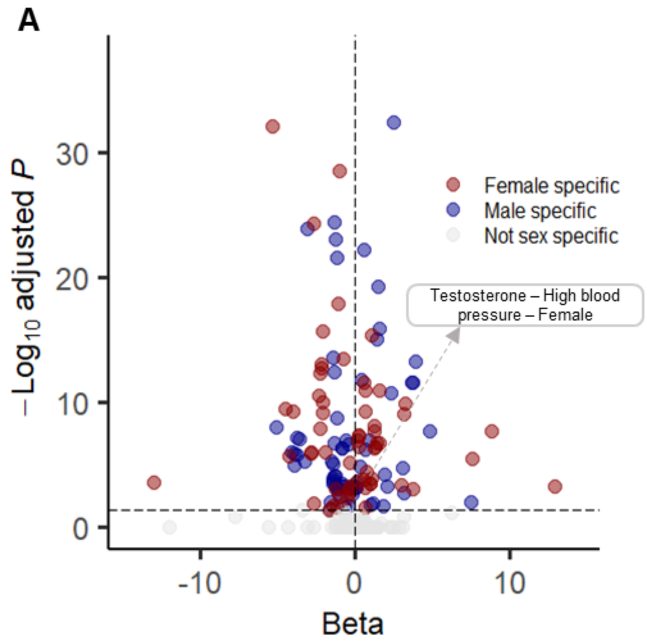

Supplementary Fig. 11. Volcano plot of sex-stratified MR test results in DMET genes region. The x-axis represents the effect size of MR-Egger test results. The y-axis represents the -log10 adjusted P-value from the MR-Egger test results. Each dot represents a MR test. The color of dots indicates whether the MR relationship is in males (blue) or females (red) or both (gray). The horizontal dashed line represents the statistical significance threshold at adjusted p= 0.05.

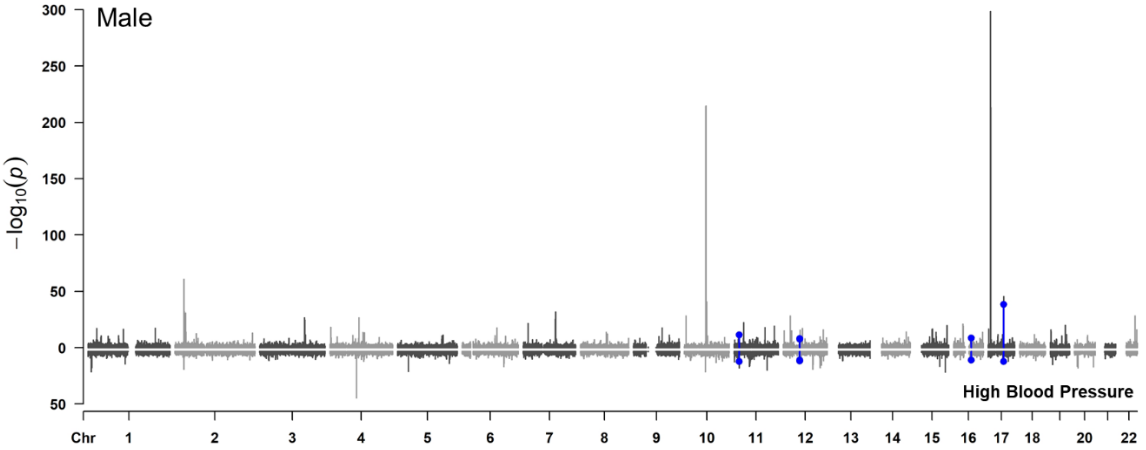

Supplementary Fig. 12. Manhattan plot for testosterone and doctor diagnosed high blood pressure in male. The x-axis corresponds to the genomic position in the genome and the y-axis to the –log10(P-value) of the GWAS results. SNPs that are statistically significant ($P < 5X10\text{-}8$) in both traits are included in MR test and highlighted in blue.

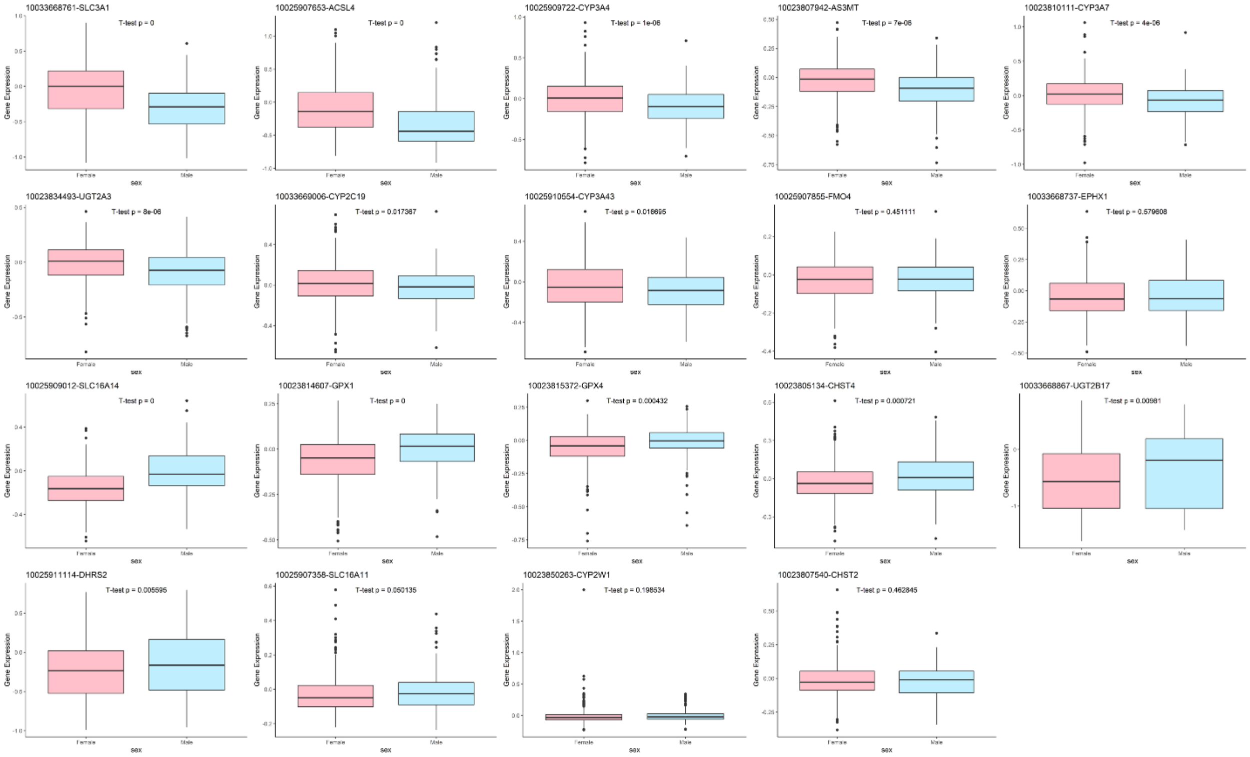

Supplementary Fig. 13. Boxplots of 19 DMET gene expression in independent validation dataset. P value of the t-test results are labeled on the plot. N_{female} = 481, N_{male} = 170. The center line of box plots in show the median, bounds of box represents the first and third quartile, whiskers extend to the minimum and maximum values within 1.5 times of the interquartile range. Two-side t-test were performed.

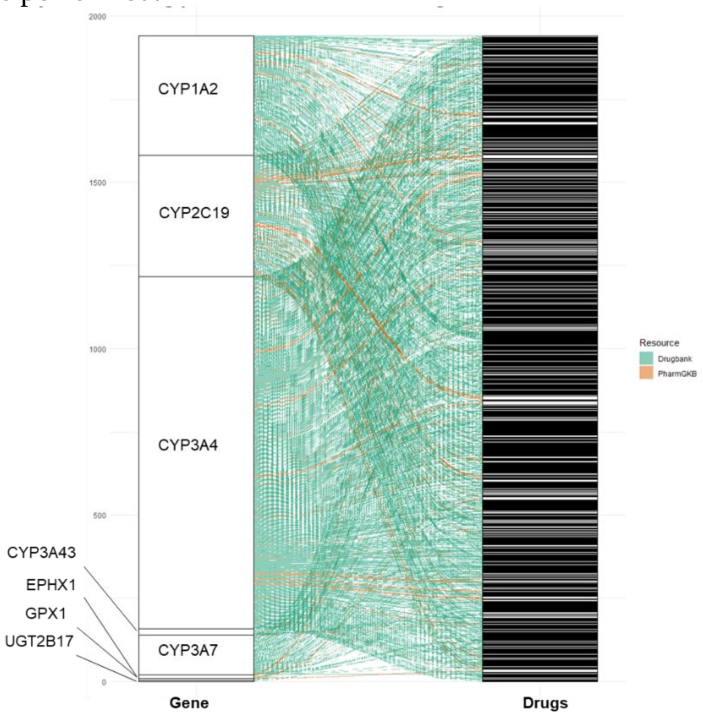

Supplementary Fig. 14. Sanky plot of drugs that annotated with sex differentially expressed genes in DrugBank and PharmGKB.

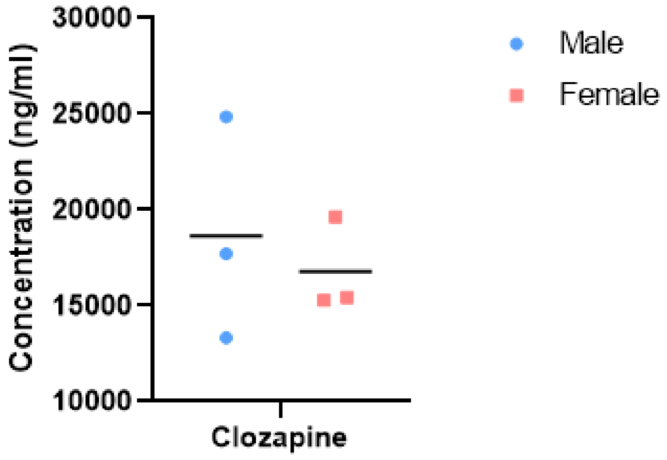

Supplementary Fig. 15. Impact of sex on clozapine metabolism in male and female pools of HLMs. Clozapine concentrations were measured by liquid chromatography-mass spectrometry (LC-MS). Each point represents an independent microsomal incubation experiment. The horizontal bar indicates the mean concentration. The color of points indicates sex (Blue: male; Red: female).

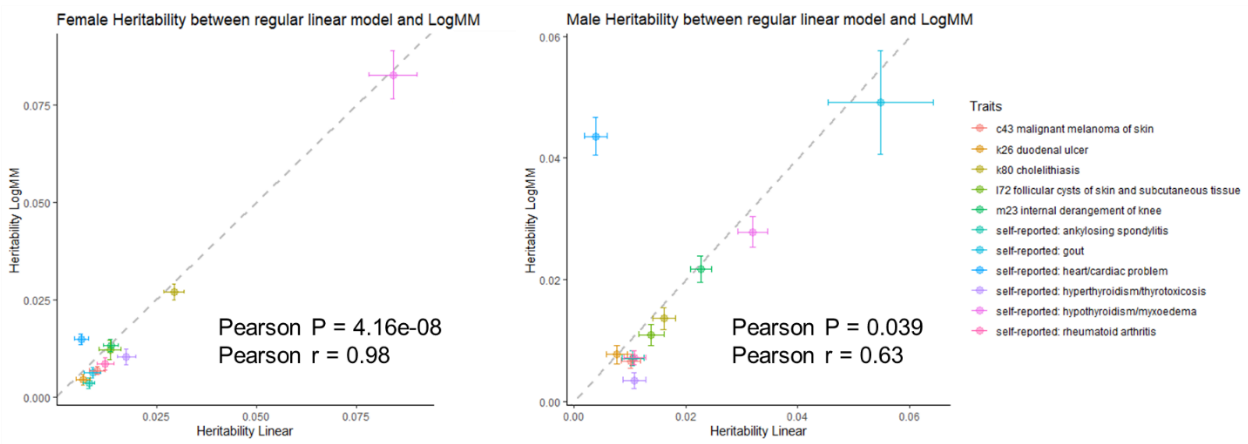

Supplementary Fig. 16. Correlation of estimated heritability of sex stratified GWAS summary statistics between regular linear association model and LogMM UK Biobank. In total 11 traits are included in this analysis. The scale bar represents the s.e. of estimated heritability. The centre indicates the value of estimated heritability.

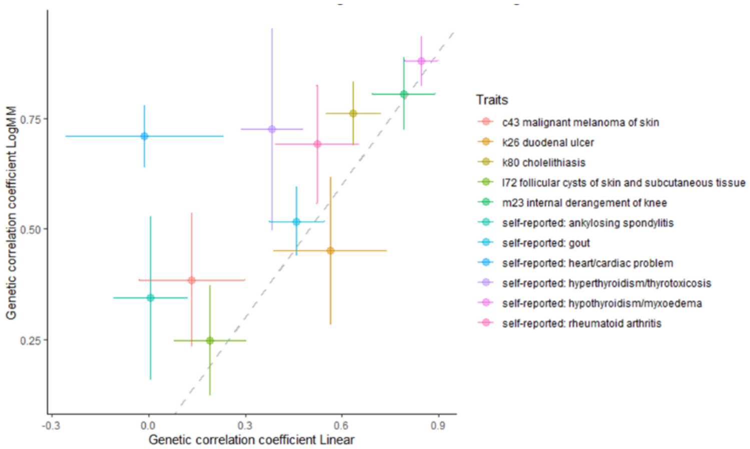

Supplementary Fig. 17. Correlation of genetic correlation coefficient in 11 traits between regular linear association model and LogMM UK Biobank. The scale bar represents the s.e. of calculated genetic correlation coefficient. The centre indicates the value of genetic correlation coefficient.

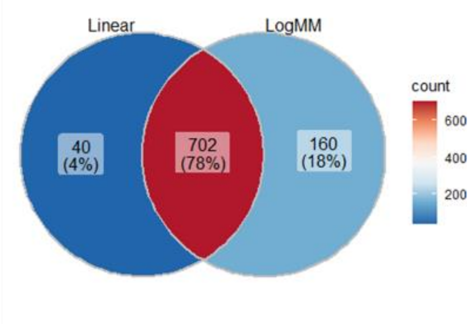

Supplementary Fig. 18. Venn diagram of sex heterogeneous SNPs identified from regular linear association model and LogMM.

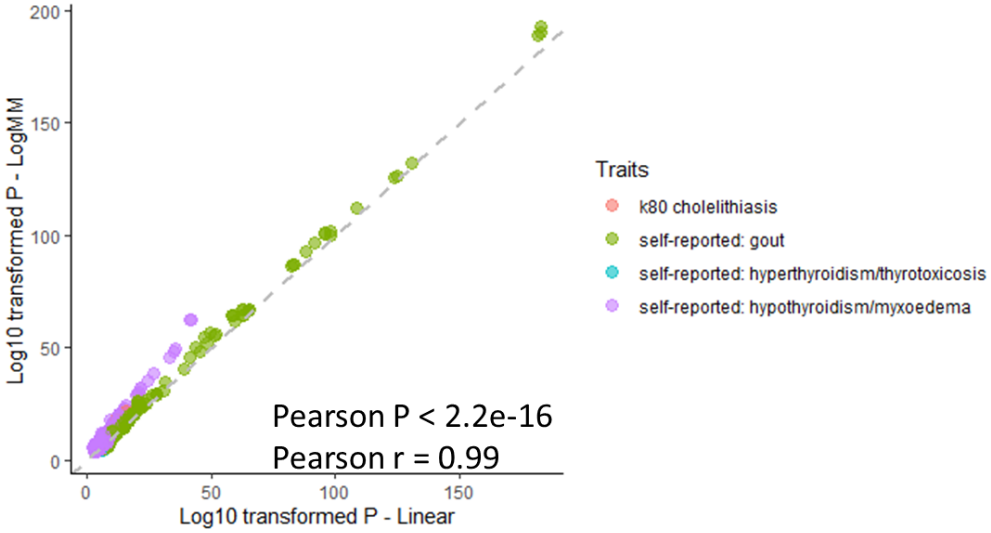

Supplementary Fig. 19. P value correlation of sex heterogenous SNPs between regular linear association model and LogMM.

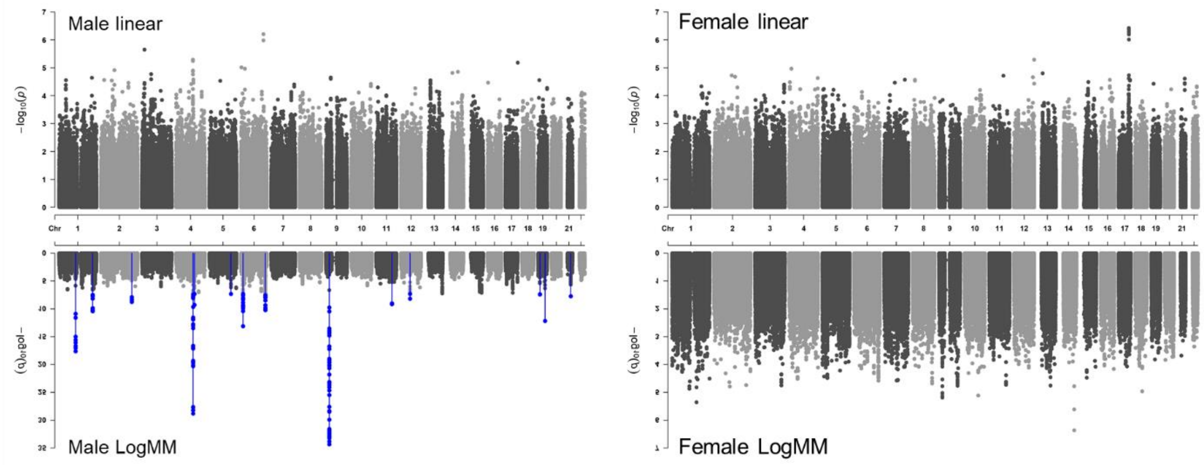

Supplementary Fig. 20. Manhattan plot of self-report heart/cardic problem in males and females. Association conducted in LogMM method is showed in the bottom. The x-axis corresponds to the genomic position in the genome and the y-axis to the –log10(P-value) of the GWAS results. Each dot represents a SNP, and SNPs that are statistically significant ($P < 5\times10^{-8}$) associated with traits are highlight in blue.
