# Supplementary material for: Deciphering genetic causes for sex differences in human health through drug metabolism and transporter genes
Source: Nat Commun. 2023 Jan 12;14:175. doi: 10.1038/s41467-023-35808-6 (PMC9837057; doi:10.1038/s41467-023-35808-6)
Supplement: Supplementary file 3 — Description of Additional Supplementary Files [file 41467_2023_35808_MOESM3_ESM.pdf]

Description of Additional Supplementary Files

File Name: Supplementary Data 1
Description: DMET genes information

File Name: Supplementary Data 2
Description: List of Traits that included in analysis

File Name: Supplementary Data 3
Description: Sex differences of esitmated heritability and genetic correlation in 564 traits

File Name: Supplementary Data 4
Description: Sex differences of esitmated heritability in 1222 nonDMET traits

File Name: Supplementary Data 5
Description: Sex differential effect of cis-DMET genes variants in 564 traits

File Name: Supplementary Data 6
Description: Colocalization of sex heterogeneous SNPs in 13 traits

File Name: Supplementary Data 7
Description: Sex stratified cis-DMET eQTL analysis

File Name: Supplementary Data 8
Description: Colocalization of sex specific cis-eQTL with binary/catagorical traits

File Name: Supplementary Data 9
Description: Colocalization of sex specific cis-eQTL with continous variables traits

File Name: Supplementary Data 10
Description: Proportion of independent SNPs mapped into cis-DMET genes region (sex stratifed & sex combined) in serum biomarker traits

File Name: Supplementary Data 11
Description: Sex differences of estimated heritability and genetic correlation in 30 serum biomarker traits

File Name: Supplementary Data 12
Description: Sex differential effect of cis-DMET genes variants in 30 serum biomarker traits

File Name: Supplementary Data 13
Description: Sex-specific causal relationships in MR test

File Name: Supplementary Data 14
Description: Sex-specific causal relationships in MR test (only cis-DMET genes SNP included)

File Name: Supplementary Data 15
Description: Sex differential DMET gene expression analysis in human liver

File Name: Supplementary Data 16
Description: Validation of 20 Sex DE genes

File Name: Supplementary Data 17
Description: Drugs annotated with sex differentially expressed genes in Drugbank and PharmGKB

File Name: Supplementary Data 18
Description: Summary of drugs reported with sex differences on PK/efficacy/toxicity

File Name: Supplementary Data 19
Description: Estimated heritability and genetic correlation of 11 traits using LogMM method

File Name: Supplementary Data 20
Description: Sex heterogeneous SNPs in 11 traits using LogMM method
