# Supplementary material for: Deciphering genetic causes for sex differences in human health through drug metabolism and transporter genes
Source: Nat Commun. 2023 Jan 12;14:175. doi: 10.1038/s41467-023-35808-6 (PMC9837057; doi:10.1038/s41467-023-35808-6)
Supplement: Supplementary file 5 — Reporting Summary [file 41467_2023_35808_MOESM5_ESM.pdf]

nature portfolio

Corresponding author(s): R.Stephanie Huang

Last updated by author(s): Dec 13, 2022

Reporting Summary

Nature Portfolio wishes to improve the reproducibility of the work that we publish. This form provides structure for consistency and transparency in reporting. For further information on Nature Portfolio policies, see our Editorial Policies and the Editorial Policy Checklist.

Statistics

For all statistical analyses, confirm that the following items are present in the figure legend, table legend, main text, or Methods section.

n/a	Confirmed	
☐	☒	The exact sample size (*n*) for each experimental group/condition, given as a discrete number and unit of measurement
☒	☐	A statement on whether measurements were taken from distinct samples or whether the same sample was measured repeatedly
☐	☒	The statistical test(s) used AND whether they are one- or two-sided<br>*Only common tests should be described solely by name; describe more complex techniques in the Methods section.*
☐	☒	A description of all covariates tested
☐	☒	A description of any assumptions or corrections, such as tests of normality and adjustment for multiple comparisons
☐	☒	A full description of the statistical parameters including central tendency (e.g. means) or other basic estimates (e.g. regression coefficient) AND variation (e.g. standard deviation) or associated estimates of uncertainty (e.g. confidence intervals)
☐	☒	For null hypothesis testing, the test statistic (e.g. *F*, *t*, *r*) with confidence intervals, effect sizes, degrees of freedom and *P* value noted<br>*Give P values as exact values whenever suitable.*
☒	☐	For Bayesian analysis, information on the choice of priors and Markov chain Monte Carlo settings
☒	☐	For hierarchical and complex designs, identification of the appropriate level for tests and full reporting of outcomes
☐	☒	Estimates of effect sizes (e.g. Cohen's *d*, Pearson's *r*), indicating how they were calculated

Our web collection on statistics for biologists contains articles on many of the points above.

Software and code

Policy information about availability of computer code

Data collection	Publications collection was done through “easyPubMed” (https://cran.r-project.org/web/packages/easyPubMed/index.html) R package.
Data analysis	The code used in this manuscript is available at Open Science Framework (OSF) and are stored in the “Decipher genetic underlying causes for sex differences in human health through the lens of drug metabolism and transporter genes” project, which can be accessed at https://osf.io/vfpjx/. Data analysis, visualization were performed in RStudio (v3.6.3)<br>HDL, v1.4.0 software is available at https://github.com/zhenin/HDL/<br>Coloc, v4 is available at https://chr1swallace.github.io/coloc/<br>TwoSampleMR, v0.5.6 is available https://mrcieu.github.io/TwoSampleMR/<br>DEseq2, v3.1.6 is available at https://bioconductor.org/packages/release/bioc/html/DESeq2.html<br>FastQTL is available at https://github.com/francois-a/fastqtl<br>LDlinkR, v1.2.2 is available at https://cran.r-project.org/web/packages/LDlinkR/index.html<br>smartSVA, v0.1.3 is available at https://cran.rstudio.com/web/packages/SmartSVA/index.html

For manuscripts utilizing custom algorithms or software that are central to the research but not yet described in published literature, software must be made available to editors and reviewers. We strongly encourage code deposition in a community repository (e.g. GitHub). See the Nature Portfolio guidelines for submitting code & software for further information.

Data

Policy information about availability of data

All manuscripts must include a data availability statement. This statement should provide the following information, where applicable:

- Accession codes, unique identifiers, or web links for publicly available datasets
- A description of any restrictions on data availability
- For clinical datasets or third party data, please ensure that the statement adheres to our policy

The UKBB GWAS summary statistics by the Neale laboratory can be obtained from http://www.nealelab.is/uk-biobank/. The summary statistics of cis-eQTL is available at the GTEx (https://gtexportal.org/home/). All GTEx protected data are available via dbGaP (phs000424.v8). Differential gene expression validation dataset is available at GSE24293 (https://www.ncbi.nlm.nih.gov/geo/query/acc.cgi?acc=GSE24293). Drug substrate information and clinical annotation for DMET genes were obtained from DrugBank (https://go.drugbank.com/releases/latest, release on 2021-01-03) and PharmGKB (https://www.pharmgkb.org/downloads, release on 2021-05-05). The LDSC-estimated heritability (https://nealelab.github.io/UKBB_ldsc/index.html). The variant effect and Combined Annotation Dependent Depletion (CADD) score were obtained from https://gnomad.broadinstitute.org/.

Human research participants

Policy information about studies involving human research participants and Sex and Gender in Research.

Reporting on sex and gender	We used "Sex" as biological attribute in our manuscript and avoid use the term of "Gender". Sex was determined based on Data-Field 31 in UK Biobank, which is acquired from central registry at recruitment. In GTEx, the Donor's Identification of sex based upon self-report, family/next of kin, or medical record abstraction. We utilized data from above 2 large consortium. The number of sample size are avaiable at http://www.nealelab.is/uk-biobank and https://gtexportal.org/home/datasets. No consent has been issued since individual data are not shared in the manuscript.
Population characteristics	No individual level data (including covariates) is available through in our study. The distribution of age, etc can be view in (https://www.gtexportal.org/home/tissueSummaryPage) for GTEx participants.
Recruitment	All GTEx individuals are deceased organ donors. Much is described in: doi: 10.1089/bio.2015.0032;<br>UK Biobank contains medical and genetic data from half a million volunteer participants
Ethics oversight	Not relevant - we do not conduct clinical study

Note that full information on the approval of the study protocol must also be provided in the manuscript.

Field-specific reporting

Please select the one below that is the best fit for your research. If you are not sure, read the appropriate sections before making your selection.

☒ Life sciences ☐ Behavioural & social sciences ☐ Ecological, evolutionary & environmental sciences

For a reference copy of the document with all sections, see nature.com/documents/nr-reporting-summary-flat.pdf

Life sciences study design

All studies must disclose on these points even when the disclosure is negative.

Sample size	The sex stratified GWAS datasets used in this study are publicly available. Detail information of sample size are available at http://www.nealelab.is/uk-biobank for GWAS summary statistics. We removed categorical/binary traits with fewer than 300 cases in either sex to reduce false discovery. The eQTL analysis were conducted after matching the sample size between males and females to maintain an equal discovery power. The differential gene expression analysis were conducted using all available RNAseq data in the GTEx-liver tissue. The drug metabolism assay were run 3 times independently.
Data exclusions	No data were excluded.
Replication	Western blot was conducted with 6 replicates in each group (Fig 4B). The clozapine metabolism assay was conducted in 3 biological replications. All attempts at replication were successful. Sex differential gene expression results were verified in an independent datasets (Fig S13). Only the clozapine metabolism assay and western blot experiments were conducted in this manuscript.
Randomization	Western blot experiments were normalized based on GAPDH abundance (Fig. 4b). Clozapine metabolism assay were conducted with negative control (No activation agents), the source data of negative control were provided in the source data file. No experiments are required for randomization/blinding design.
Blinding	Not relevant - no experiments are required for randomization/blinding design

Reporting for specific materials, systems and methods

We require information from authors about some types of materials, experimental systems and methods used in many studies. Here, indicate whether each material, system or method listed is relevant to your study. If you are not sure if a list item applies to your research, read the appropriate section before selecting a response.

Materials & experimental systems

n/a	Involved in the study
☐	☒ Antibodies
☒	☐ Eukaryotic cell lines
☒	☐ Palaeontology and archaeology
☒	☐ Animals and other organisms
☒	☐ Clinical data
☒	☐ Dual use research of concern

Methods

n/a	Involved in the study
☒	☐ ChIP-seq
☒	☐ Flow cytometry
☒	☐ MRI-based neuroimaging

Antibodies

Antibodies used	All antibodies were purchased from ABclonal including<br>CYP1A2 Rabbit pAb: A0062, CYP3A4 Rabbit pAb: A2544, GAPDH Rabbit mAb: A19056, clone.No ARC50882. HRP Goat Anti-Rabbit IgG (H+L): AS014
Validation	Validation of all primary antibodies for the species and application was conducted by manufacturers prior to sale, and validation statements are available on the manufacturers' website. The GAPDH Rabbit mAb used in this study was validated in PMID: 34666787; The CYP1A2 Rabbit pAb used in this study was validated in PMID: 33965855; The CYP3A4 Rabbit pAb used in this study was validated in PMID: 28726775; The HRP Goat Anti-Rabbit IgG (H+L)used in this study was validated in PMID: 26423004.
